# Frontiers of nanoelectronics: intrinsic Josephson effect and prospects of superconducting spintronics

**DOI:** 10.3762/bjnano.14.9

**Published:** 2023-01-10

**Authors:** Anatolie S Sidorenko, Horst Hahn, Vladimir Krasnov

**Affiliations:** 1 Institute of Electronic Engineering and Nanotechnologies of the Technical University of Moldova, Academiei 3/3, Chisinau 2028, Moldova; 2 I.S. Turgenev Orel State University, Komsomolskaya str. 95, 302026, Orel, Russia; 3 Institute of Nanotechnology, Karlsruhe Institute of Technology (KIT), D-76021 Karlsruhe, Germany; 4 Chemical, Biological and Materials Engineering, The University of Oklahoma, Norman, 73019, OK, United States; 5 Department of Physics, Stockholm University, AlbaNova University Center, SE-10691 Stockholm, Sweden

**Keywords:** artificial neural networks, functional nanostructures, intrinsic Josephson effect, nanoelectronics, spintronics

The twenty-first century is marked by an explosive growth in the flow of information, which is necessary to process, archive, and transmit data through communication systems. For that purpose, big data centers with powerful supercomputers have been created all over the world, consuming a huge amount of electricity. For example, just one of thousands of big data centers worldwide, located in the town of Lulea, Sweden [1] consumes 9% of the electricity of the entire country. On the other hand, during the last four decades, the triumphal development of microelectronics and computers, based on traditional semiconductor chips, was enabled by the exponential growth of the number of transistors in chips and the shrinkage of the size of individual transistors, following the empirical Moore’s Law, which is now showing slowing-down and failure signs [2].

It is evident that a radical reduction in energy consumption through efficiency improvement is needed and has become one of the main goals in the development of new supercomputers. For example, the powerful modern supercomputer TIANHE-2, a massive system that clocked 33.86 petaflops (i.e., 33.86 thousand trillion floating-point operations per second) has a power requirement of 17.6 MW (taking into consideration the external cooling, the power requirement is 24 MW) [3], which is comparable to the power requirement of a city.

To overcome these problems concerning increasing energy demands, a revolutionary solution is needed with two goals to be simultaneously reached: energy saving and increase in the capability of novel computers. The future of high-performance computing with low energy consumption is clearly associated with technologies with drastically lower energy dissipation.

А logical solution and the most promising candidate for radical reduction in energy consumption is the superconducting digital technology (SDT) based on Josephson junctions. The intrinsic Josephson effect, which was first reported by Reinhold Kleiner, Paul Müller, and co-workers (see [4–5] and references therein) has been investigated by many researchers [6–8]. The energy consumption of the SDT basic element is of the order of 10^−19^ J, corresponding to up to seven orders of magnitude less energy dissipation than that for their semiconductor analog, even when the energy for cryogenic cooling of superconducting circuits is considered [9–12]. Important and promising applications of the Josephson effect are its implementation in superconducting high-frequency electronics, spintronics, and nanostructures for supercomputers. In the last decade, a very rapid development in superconducting spintronics, based on functional nanostructures and Josephson junctions, has taken place [13–14]. The implementation of such devices in building blocks for quantum computers and for novel computers using non-von Neumann architecture with brain-like artificial neural networks (ANN) is a recent important development.

The main goal of this thematic issue is to highlight new research done in the field of hybrid nanostructures, including various elements using the Josephson effect and their applications in quantum electronics, spintronics, and high-frequency electronics.

The following novelties are presented in the contributed articles of this volume:

- Novel promising spintronic elements and materials with controllable switching between stable parallel, orthogonal, and antiparallel arrangements of magnetic moments of the epitaxial PdFe films and PdFe/Ag/PdFe heterostructures [15–16].

- Detection of ultrahigh frequency radiation by new devices:

- Based on Josephson junctions with frequencies of 72–265 GHz using the Josephson grain boundary junction fabricated in YBaCuO films [17] and broad-band detectors based on YBaCuO Josephson junctions fabricated on ZrYO bicrystals with a very high responsivity at 77 K (up to 9 kV/W), low noise equivalent power (NEP) of 3 × 10^−13^ W/Hz^(1/2)^, and with a wide power dynamic range equal to 1 × 10^6^ [18].
- Integrating an aluminum Josephson junction, with a size of a few micrometers, operating as a single photon counter in the microwave frequency range, switching from the superconducting to the resistive state due to absorption of a 10 GHz external signal [19].
- Achieving a large coherent gain of the receiver, up to a factor of three, of the emitted power from two simultaneously biased arrays of Josephson junctions compared to that of the sum of the power values from two individually biased arrays. The detected phenomenon is attributed to the phase locking of Josephson junctions in different arrays via a common electromagnetic field [20].
- Modeling of a multi-frequency receiving system based on an array of dipole antennas with cold-electron bolometers, with photon NEP of 1.1 × 10^−16^ W/Hz^(1/2)^, achieved by replacing one of two single superconductor–insulator–normal (SIN) tunnel junctions with a single superconductor–normal (SN) contact [21].
- Proposing a new type of cold electron bolometers with traps and hybrid superconducting/ferromagnetic absorber with a temperature reduction of the electrons in the refrigerator junctions down to 25 mK in the idle regime without an optical power load [22].

- Implementation of Josephson junctions for the design of quantum computers by analyzing the dynamics of a single-junction superconducting interferometer as an adiabatic neural cell of a perceptron artificial neural network in the quantum regime (a hybrid system whose configuration is dynamically adjusted by a quantum co-processor) [23].

- In several articles of the volume, new phenomena are predicted and investigated for promising spintronics applications, such as:

- Plasma modes in capacitively coupled superconducting nanowires have been predicted [24]. It has been demonstrated that in the presence of inter-wire coupling plasma modes, each of the modes get split into two "new" modes propagating with different velocities across the system.
- The magnetic proximity effect at a ferromagnetic–insulator–superconductor (FIS) interface was investigated through combined experimental and theoretical work [25].
- Manifestations of nonlinear features in magnetic dynamics and current–voltage characteristics of the 0 Josephson junction in superconductor–ferromagnet–superconductor (SFS) structures have been predicted and calculated [26].
- A quantitative study of the density of states (DOS) in bulk superconductor/ferromagnetic (S/F) bilayers in the diffusive limit has been presented. In addition, an analysis of the dependencies of DOS on magnetic and spin–orbit scattering times has been carried out [27].

In addition, the volume also highlights some other interesting effects in S/F functional nanostructures, which may be implemented in future spintronics devices.

The concept of this thematic issue was developed during the International SPINTECH Conference “NANO-2021: The 12th International Conference on Intrinsic Josephson Effect and Horizons of Superconducting Spintronics”, which took place in September 2021 in Chisinau, Moldova. The presentations of the participants of the conference incorporating new ideas, technological approaches for the design of functional nanostructures for superconducting spintronics, quantum electronics, and novel base elements for superconducting supercomputers became the core of this volume.

The editors are convinced that this thematic issue will attract the attention of scientists, technologists, engineers, and IT experts, and will be useful for a broad readership including students wishing to extend their knowledge in this new, rapidly developing, and highly promising area.

Anatolie S. Sidorenko, Horst Hahn, and Vladimir Krasnov

Chisinau, Karlsruhe, and Stockholm, December, 2022.
